# Schiff Base–Derived Colorimetric Chemosensor for Fe^3+^ Detection: Synthesis, Mechanistic Insights, and Real‐World Applications in Water Quality Monitoring

**DOI:** 10.1155/ianc/3977293

**Published:** 2026-02-23

**Authors:** Shahad Ayed Alahmady, Syed Nazreen, Ali Q. Alorabi

**Affiliations:** ^1^ Department of Chemistry, Faculty of Science, Al-Baha University, Al-Baha, 65779, Saudi Arabia, bu.edu.sa

**Keywords:** binding stoichiometry, colorimetric detection, Fe^3+^ detection, paper strip detection, Schiff base

## Abstract

A Schiff base colorimetric chemosensor (H_2_L), synthesized from 2‐hydroxy‐1‐naphthaldehyde and anthranilic acid, was developed for selective Fe^3+^ detection. In a DMF:H_2_O (9:1, v/v) medium, H_2_L exhibited a distinct and selective response to Fe^3+^ among common competing cations, producing a visible color change from yellow to black and a new broad absorption band at 504 nm. Job’s plot indicated a 1:1 binding stoichiometry, and the association constant was 2.87 × 10^4^ M^−1^. The sensor showed a low detection limit of 3.71 μM by UV–Vis titration, and its practical applicability was validated by spike–recovery analysis in real water samples (recoveries 91.04%–100.94%). A paper‐strip assay enabled rapid on‐site screening with smartphone image analysis, achieving an LOD of 50.7 μM. These results highlight H_2_L as a simple and effective platform for Fe^3+^ monitoring in water‐relevant matrices.

## 1. Introduction

Heavy metal contamination presents a significant global threat due to their severe toxicity to human health and the environment [1]. The high stability and solubility of many metal species in aquatic systems promote their persistence and accumulation, particularly in industrial effluents originating from mining, pesticide production, battery manufacturing, metal smelting, fertilizer processing, and the pulp and paper industry [2–4]. Among these metals, iron (Fe^3+^) plays vital roles in biological systems, contributing to processes such as deoxyribonucleic acid and ribonucleic acid synthesis, electron transport, oxygen metabolism, enzymatic activity, and cellular respiration [5, 6]. However, both iron deficiency and overload are associated with serious health conditions, including anemia, asthma, kidney and liver damage, diabetes, and cardiovascular diseases [7–9]. Therefore, precise detection of Fe^3+^ in aqueous media is critical, which necessitates new sensor fabrication.

Therefore, scientists have developed various sophisticated techniques for the determination of Fe^3+^ in water matrices, which remains essential and motivates the development of reliable sensing platforms. Conventional methods for iron analysis include atomic absorption spectrometry, voltammetry, and inductively coupled plasma [10, 11]. While these techniques are superlative, they require expensive instruments, skilled operators, and pretreatment procedures [12, 13]. In this context, chemosensor‐ and fluorescence‐based methods have emerged as attractive alternatives due to their operational simplicity, rapid response, and potential for selective detection of biologically relevant metal ions [14–16]. More recently, chemosensor research has increasingly emphasized portability, reversibility, and stronger mechanistic validation. For instance, smartphone‐assisted analytical platforms enable real‐time, on‐site detection using image‐based quantification, highlighting the growing emphasis on portability and instrument‐free readout [17]. In parallel, reversible Schiff‐base chemosensors operating in both solution and solid state have been explored to enhance practicality and reusability [18]. Moreover, many contemporary studies integrate spectroscopic titrations with computational analyses to support binding modes and signal‐transduction mechanisms, thereby strengthening interpretability and credibility [19]. Similarly, ratiometric and dual‐mode Schiff‐base systems have been developed for transition‐metal sensing, illustrating how rational ligand design can improve selectivity and analytical robustness [20]. Collectively, these developments provide an updated framework for positioning Fe^3+^ colorimetric probes intended for water‐relevant matrices.

Recent review‐level contributions further emphasize the continued growth of Fe^3+^ sensing and Schiff‐base design. Rhodamine‐based Schiff‐base chemosensors have been comprehensively discussed for metal‐ion detection, including Fe^3+^‐responsive systems, with emphasis on structure–response relationships and practical optical readouts [21]. In addition, recent summaries of organic chemosensors (including pyrazoline and related frameworks) underscore current selectivity strategies for Fe^3+^ and competing ions in solution [22]. In parallel, alternative signal platforms such as carbon dots have been reviewed for Fe^3+^/Fe^2+^ determination, reflecting ongoing efforts toward sensitivity enhancement and field applicability [23].

Accordingly, numerous chemosensors have been developed for Fe^3+^ detection, such as rhodanine azo dyes [24], benzo‐imidazo‐pyrrolo [3,4‐c] pyridines [25], coumarin‐incorporated fluorogenic probes [26], naphthalene‐immobilized nanoporous silica [27], thiacalix [4] arene [28], amidine‐based chemosensors [29], and rhodamine‐based hexapodal structures [30]. Among the many ligand families explored, Schiff bases (R_1_R_2_C = NR′(R′ ≠ H)) have garnered significant attention due to their synthesis simplicity, excellent coordination ability, and crucial pharmacological properties, including anticancer, antifungal, antibacterial, and anti‐inflammatory activities [31–35]. Their thermal stability and complex‐forming capability further make Schiff bases ideal candidates for metal ion sensing, including Fe^3+^ [7, 36–43]. For instance, You et al. [36] synthesized a Schiff‐base sensor from 2‐(2‐aminoethoxy)ethanamine moiety with 2,3‐dihydroxybenzaldehyde, achieving an Fe^3+^ detection limit below the World Health Organization (WHO) permissible level in drinking water (5.37 μM). Likewise, Singh et al. [38] developed a Schiff‐base–chalcone‐functionalized triazole bis‐organosilane sensor with excellent selectivity and sensitivity toward Fe^3+^ ions.

Based on those considerations, a Schiff base–derived colorimetric chemosensor (2‐((2‐hydroxynaphthalen‐1‐yl) methyleneamino) benzoic acid) (H_2_L), was synthesized, leveraging its unique chemical structure for metal ion detection. To the best of our knowledge, H_2_L has not yet been investigated as a chemosensor for the detection and quantification of metal ions. Accordingly, this study aims to synthesize and spectroscopically characterize H_2_L (FTIR, NMR, and ESI–MS), evaluate its sensing capabilities and selectivity toward Fe^3+^, determine binding behavior and conditions, and demonstrate practical applicability through spike–recovery analysis in real water samples and a paper‐strip format suitable for rapid on‐site screening.

## 2. Experiments

### 2.1. Materials and Instruments

All materials used were of analytical or spectroscopic grade, supplied commercially, and used without additional treatments. Anthranilic acid, 2‐hydroxy‐1‐naphthaldehyde, and metal salts—including aluminum(III) chloride hexahydrate (AlCl_3_·6H_2_O; 99%), barium(II) chloride dihydrate (BaCl_2_·2H_2_O; 99%), calcium(II) chloride dihydrate (CaCl_2_·2H_2_O; 97%), cobalt(II) chloride hexahydrate (CoCl_2_·6H_2_O; 98%), chromium(III) chloride hexahydrate (CrCl_3_·6H_2_O; 93%), copper(II) chloride dihydrate (CuCl_2_·2H_2_O; 99%), iron(III) chloride anhydrous (FeCl_3_; 99%), mercury(II) chloride (HgCl_2_; 99.5%), potassium chloride (KCl; 99.5%), magnesium(II) chloride hexahydrate (MgCl_2_·6H_2_O; 98%), manganese(II) chloride tetrahydrate (MnCl_2_·4H_2_O; 97%), sodium chloride (NaCl; 99.5%), nickel(II) chloride hexahydrate (NiCl_2_·6H_2_O; 98%), lead(II) chloride anhydrous (PbCl_2_; 98%), and zinc nitrate hexahydrate (Zn(NO_3_)_2_·6H_2_O; 98%)—were procured from Lobachemie (Mumbai, India). Absolute ethanol (EtOH; ≥ 99.5%), methanol (MeOH; ≥ 99.8%), acetonitrile (CAN; 99.9% by GC), dimethyl sulfoxide (DMSO; > 99.9%), and dimethylformamide (DMF; ≥ 99.8%) were obtained from Fisher Scientific (MA, USA).

The chemical structures of H_2_L and L–Fe(III) were affirmed using various spectrometric techniques. FTIR spectra were acquired using a Nicolet iS50 FTIR (Thermo Nicolet Corporation, Waltham, MA, USA) following the KBr‐disc technique within the 400–4000 cm^−1^ range. NMR patterns were obtained in DMSO‐d6 solvents using a 500 MHz NMR spectrometer (Bruker, JEOL, Tokyo, Japan), with TMS used as an internal reference. Ultraviolet–visible (UV–Vis) spectra were recorded using an Evolution 201 instrument (Thermo Scientific, Melbourne, Australia); baseline correction was performed using a matrix‐matched blank (DMF:H_2_O, 9:1, v/v), and for differential sensing plots, the H_2_L solution in the same medium was used as the reference blank. Mass spectra (MS) were acquired using the electrospray ionization (ESI) technique on a Thermo Scientific LCQ‐FLEET spectrometer (Thermo Fisher Scientific, Waltham, MA, USA).

### 2.2. Synthesis of Schiff Base Ligand (H_2_L)

H_2_L was synthesized following the reported procedure [44]. Briefly, equimolar amounts of 2‐hydroxy‐1‐naphthaldehyde (8.60 g, 0.05 mol) and anthranilic acid (6.85 g, 0.05 mol) were dissolved in EtOH (50 mL), and the mixture was refluxed under continuous stirring for 6 h. The reaction progress was monitored by thin‐layer chromatography. After completion, the solvent volume was reduced to approximately 20 mL by evaporation, and the solution was allowed to cool to room temperature to afford a yellow precipitate. The solid was collected by filtration, washed with EtOH, recrystallized from EtOH, and dried to give H_2_L in 90% yield.

### 2.3. Synthesis of L–Fe(III) Complex

**FIGURE 1:**
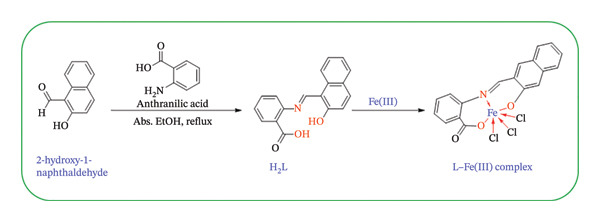
Synthesis of H_2_L ligand and its L–Fe(III) complex.

The Fe(III) complex was prepared by reacting H_2_L (1.0 mmol) with FeCl_3_·6H_2_O (1.0 mmol). H_2_L was dissolved in EtOH (20 mL) under continuous stirring. Separately, FeCl_3_·6H_2_O (1.0 mmol) was dissolved in EtOH (20 mL), and resulting solution was added dropwise to the ligand solution. The mixture was refluxed under stirring for 6 h. After completion, the precipitate was collected by filtration, washed thoroughly with EtOH followed by diethyl ether to remove residual reactants, and dried under vacuum at room temperature. The proposed reaction scheme is shown in Figure 1.

### 2.4. Colorimetric Sensing for Fe(III)

#### 2.4.1. Solvent Selection

The solubility of H_2_L was evaluated in four solvents: AcN, DMF, DMSO, and MeOH to identify the most suitable solvent for subsequent experiments. For each solvent, a 10 mM stock solution was prepared by dissolving 0.029 g of H_2_L in 10 mL of the respective solvent, and solubility was assessed visually.

#### 2.4.2. Effect of Water on H_2_L Solubility

The solubility of H_2_L was further examined in DMF:H_2_O mixtures with varying water content (10%–90% H_2_L, v/v). Typically, 1 mL of H_2_L stock solution (10 mM in DMF) was added to volumetric flasks containing 0–9 mL of deionized water, and the final volume was adjusted to 10 mL, yielding a 1.0 mM H_2_L solution. The solubility of H_2_L in each mixture was then visually assessed. Additionally, the effect of the DMF:H_2_O composition on the absorbance intensity of the L–M^n+^ complex was investigated. For this purpose, aqueous solutions (10 mM) of testing metal ions, including Al^3+^, Ba^2+^, Ca^2+^, Co^2+^, Cr^3+^, Cu^2+^, Fe^3+^, Hg^2+^, K^+^, Mg^2+^, Mn^2+^, Na^+^, Ni^2+^, Pb^2+^, and Zn^2+^, were prepared in deionized water and subjected to UV–Vis analysis in the presence of H_2_L under varying solvent conditions.

### 2.5. pH Effect Studies

The influence of pH on the L–Fe(III) complex formation was investigated by adjusting the pH using dilute HCl and NaOH solutions. A reaction mixture was prepared by combining 5 mL of the H_2_L stock solution (10 mM), 0.5 mL of the Fe(III) stock solution (10 mM), and 40 mL of DMF in a 50 mL measuring flask. The desired concentrations were achieved by dilution with distilled water. UV–Vis absorption spectra were recorded across different pH values to evaluate the pH‐dependent behavior of the complex.

### 2.6. Colorimetric Analysis

The colorimetric response of H_2_L toward various metal ions was evaluated using UV–Vis spectroscopy. Therefore, 1 mL of H_2_L stock solution (10 mM) and 8 mL of DMF were added to 10 mL flasks containing 1 mL of the corresponding metal ion solution (10 mM). The final concentrations of both reactants were standardized to 1 mM. Absorption measurements were subsequently recorded to assess the interaction between H_2_L and each metal ion.

### 2.7. Job’s Plot Method–Coordination Stoichiometry

The binding stoichiometry was determined using Job’s plot method of continuous variation. A series of nine solutions was prepared by mixing varying volumes of Fe(III) (0.9–0.1 mL) and H_2_L (0.1–0.9 mL) while keeping a constant total molar concentration. The total volume of each solution was adjusted to 10 mL using a DMF:H_2_O mixture (9:1, v/v). UV–Vis absorbance measurements were recorded at *λ*_max_ = 504 nm, and a plot of absorbance versus the mole fraction of Fe(III) was constructed.

### 2.8. Titration Experiments: Limit of Detection (LOD) and Association Constant

The LOD and limit of quantification (LOQ) of Fe(III) by H_2_L were determined based on their interaction at varying Fe(III) concentrations (10–1000 μM). Working solutions were prepared by adding Fe(III) to 10 mM H_2_L and adjusting the total volume to 10 mL with DMF:H_2_O (9:1, v/v). The absorbance values at 504 nm were plotted against Fe^3+^ concentrations using UV–Vis spectroscopy. The LOD and LOQ values were calculated using Equations (1) and (2), respectively.

(1) LOD=3.3×δS,

(2) LOQ=3.3×δS,

where *δ* is the standard deviation and *S* is the slope of the analyzed data [45, 46].

Moreover, the association constant of L–Fe(III) binding was calculated using the Benesi–Hildebrand formula [47] as follows:

(3) DoAbs=1Ao1εka+1ε,

where [D]_o_, [A]_o_, Abs, *ε*, and ka are the ligand concentration, metal concentration, complex absorbance, molar absorptivity, and association constant, respectively. The binding constant was obtained from a plotted 1/[A]_o_ versus [D]_o_/Abs.

### 2.9. Interferences With Other Metal Ions

The potential interference of competing metal cations on the formation of the L–Fe(III) complex was evaluated in a DMF:H_2_O solvent mixture (9:1, v/v). For each test, 1 mL of H_2_L solution (10 mM) and 0.5 mL of Fe(III) solution (10 mM) were mixed with 0.5 mL of a pre‐prepared metal ion solution (10 mM), including Zn^2+^, Cr^3+^, Ni^2+^, Mn^2+^, K^+^, Hg^2+^, Co^2+^, Mg^2+^, Ca^2+^, Ba^2+^, Pb^2+^, Na^+^, Al^3+^, and Cu^2+^. The mixture was adjusted to 10 mL with DMF, yielding a 1 mM H_2_L concentration. A control solution containing only H_2_L and Fe(III) (1 mL of 10 mM stock solutions) diluted to 10 mL with DMF was also prepared for comparison. All solutions were analyzed using UV–Vis spectroscopy at 504 nm.

### 2.10. Detection of Fe(III) in Real Water Samples

The applicability of H_2_L for Fe(III) detection was assessed using three real water samples: (i) AlAqiq Dam (Al‐Baha Province); (ii) distilled water; and (iii) tap water from Al‐Baha University. Each sample (1 mL) was spiked with varying volumes (0, 100, 200, and 300 μL) of Fe(III) stock solution (10 mM). Subsequently, 1 mL of H_2_L stock solution (10 mM) was added and diluted to 10 mL with DMF, yielding Fe(III) concentrations ranging from 0.0 to 0.3 mM, while maintaining a constant H_2_L concentration of 1.0 mM. The sensor’s performance was then evaluated using UV–Vis spectra.

#### 2.10.1. Paper Strip Detection and Analysis

Whatman Grade 1 filter paper was cut into strips (1.5 × 10 cm) to fabricate paper‐based sensors. These strips offer a rapid, on‐site method for Fe(III) detection without complicated steps or significant user involvement. The paper stripes were immersed in H_2_L solution (10 mM) for 1–2 min and air‐dried at room temperature for 24 h to ensure uniform deposition of the chemosensor H_2_L (Figure S1). For colorimetric analysis, the prepared paper strips were observed visually after exposure to Fe^3+^ ions. Upon Fe^3+^ complexation, the color of H_2_L on the test strips gradually changed from orange to brown (Figure S2). To quantify these colorimetric changes, test strip images were taken using a smartphone camera. The images were then processed by inverting colors using an online tool (https://pinetools.com/invert-image-colors) and analyzed with ImageJ software (National Institute of Health, USA), following a previously established protocol [48]. For image analysis, the red, green, and blue (RGB) channels were examined, with the blue channel providing the highest intensity. Measurement areas were defined by drawing squares within the reaction zone of the test strips. The “Measure” function under the “Analyze” tab in ImageJ was used to obtain color intensity values. Each test strip was measured 20 times, and the average color intensity was calculated using Microsoft Excel, with unexposed H_2_L strips serving as blanks. The color intensity difference between H_2_L test strips and L–Fe(III) test strips was used to quantify Fe(III) detection. Origin 18 software was used for drawing and calculation of the slope, coefficient of determination (*R* ^2^), and standard deviation.

## 3. Results and Discussion

### 3.1. Characterization

#### 3.1.1. FTIR Analysis

**FIGURE 2:**
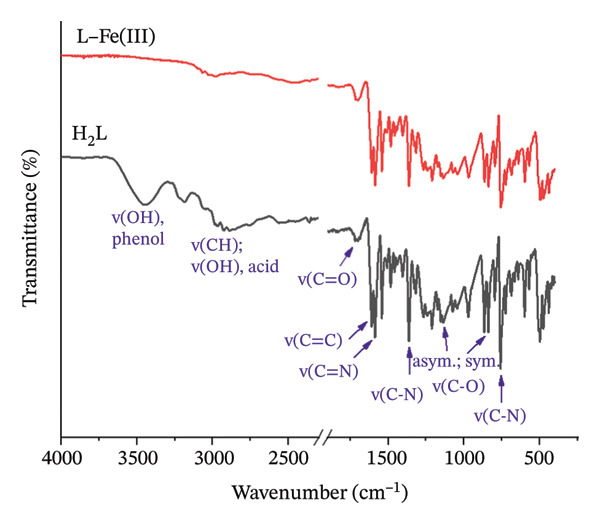
FTIR spectra of H_2_L ligand and its L–Fe(III) complex.

The FTIR spectra of H_2_L and L–Fe(III) were depicted in Figure 2. In the H_2_L spectrum, the peaks corresponding to phenolic and carboxylic O‐H stretching vibrations appear as broad bands centered at approximately 3445 and 2850 cm^−1^ [49]. However, the disappearance of these bands upon complexation confirms the successful coordination with Fe^3+^ ions. The characteristic peaks at 1709, 1606, 1585, 1546, and 1364 cm^−1^, attributed to C=O, C=C, −CH=N−, COO^−^, and C‐N vibrations, respectively [44, 50, 51], exhibited slight variations, further supporting the coordination of Fe^3+^ with azomethine nitrogen (−CH=N−) and the deprotonated carboxylic and phenoxide groups. Additionally, the peaks observed in the ranges of 1315–1135 cm^−1^ and 970–865 cm^−1^ correspond to the asymmetric and symmetric vibrational modes of C‐C‐OH. The peak at 865 cm^−1^ in H_2_L was slightly shifted to 860 cm^−1^ in the complex spectrum, further confirming the coordination process.

#### 3.1.2. NMR Analysis

The ^1^H NMR spectrum of H_2_L revealed a singlet peak at *δ* 8.88 ppm, corresponding to the azomethine linker (−CH=N−) (Figure 3(a)), which was further supported by a peak at *δ* 151.58 ppm in the ^13^C NMR spectrum (Figure 3(b)) [52]. The aromatic region appeared between *δ* 6.74 and 8.34 ppm, while the OH and COOH protons were observed at *δ* 10.77 and 11.96 ppm, respectively. As shown in Figure 3(b), the formation of H_2_L was further corroborated by the downfield signals in the ^13^C NMR at *δ* 176.45 and 167.88 ppm, corresponding to the carbonyl and C‐OH carbons, respectively. The ^1^H NMR (Figure 3(c)) and ^13^C NMR (Figure 3(d)) spectra of the L–Fe(III) complex were comparable to those of H_2_L. However, the ^1^H NMR of the complex displayed a downfield shift of the CH=N–proton from *δ* 8.88 to 8.93 ppm, indicating coordination of the azomethine nitrogen to Fe(III) and electron delocalization toward the metal ion. Additionally, the disappearance of OH proton signals in the complex suggested deprotonation during complex formation. The ^13^C NMR spectrum of the complex showed no appreciable changes in most peaks upon coordination, except for a downfield shift of the azomethine carbon signal to *δ* 169.10 ppm.

FIGURE 3
^1^H NMR (a, c) and ^13^C NMR (b, d) spectra of H_2_L ligand and L–Fe(III) complex, respectively.

**Figure:**
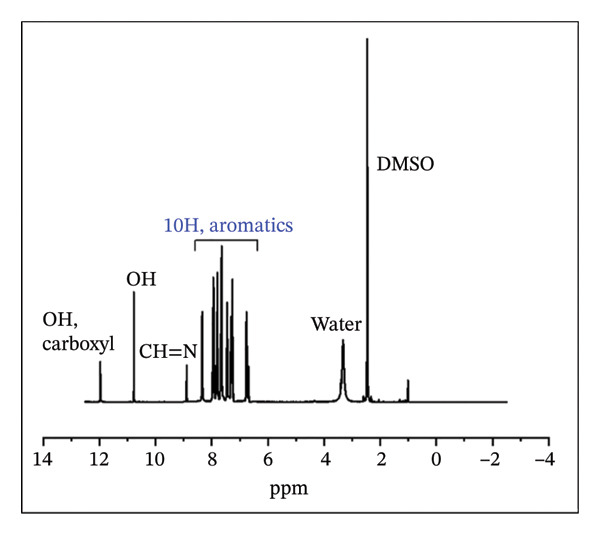
(a)

**Figure:**
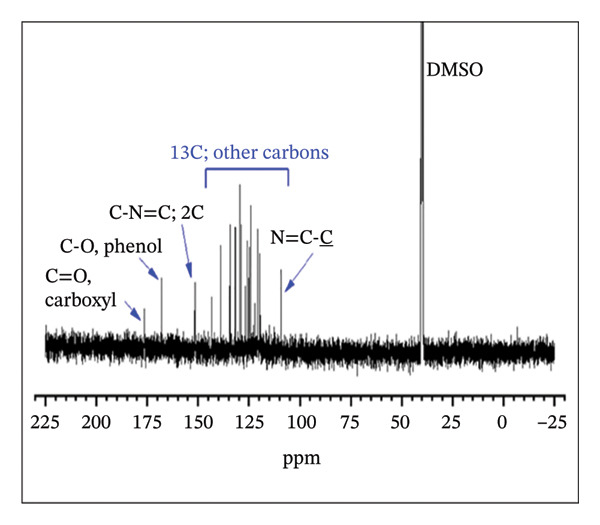
(b)

**Figure:**
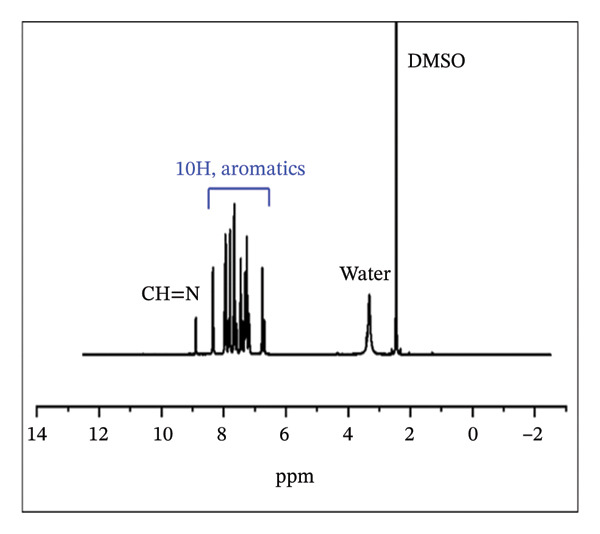
(c)

**Figure:**
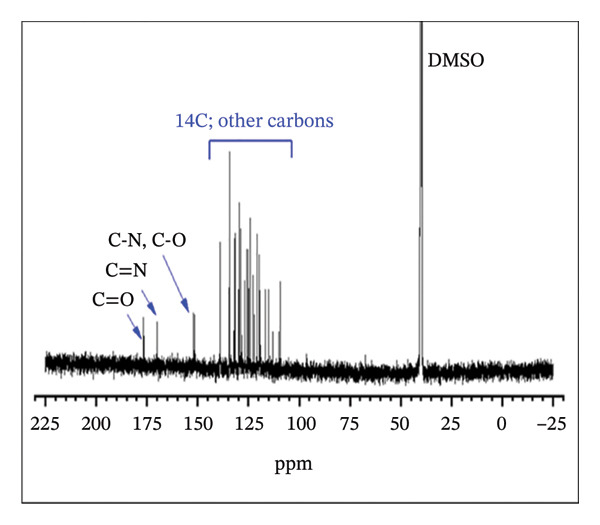
(d)

#### 3.1.3. MS

The chemical structures of the free ligand (H_2_L) and its Fe(III) complex were further examined by ESI–MS. The mass spectrum of H_2_L exhibited peaks at m/z of 291, 290, and 246, corresponding to the molecular ion [M], the H‐truncated fragment, and the carboxyl‐truncated fragment, respectively (Figure S3a). For the Fe(III) system, the ESI–MS spectrum (Figure S3b) is dominated by ligand‐derived fragments, while a signal attributed to a metal–ligand species appears at lower relative abundance (∼5%). This can occur for metal–ligand adducts under electrospray conditions due to partial in‐source dissociation and competing adduct formation; therefore, the ESI–MS data are reported as complementary confirmation consistent with Fe(III) complexation, in agreement with the UV–Vis response, Job’s plot, and other spectroscopic results.

A diagnostic ion at m/z 397.42 (fragment A, Figures S3 and S4) is consistent with a chloro‐containing Fe–ligand adduct and was tentatively assigned as [Fe(L)Cl+NH_4_]^+^, where L denotes the deprotonated ligand. Because Fe(III) readily undergoes ligand exchange between chloride and solvent molecules, the participation of chloride in the inner coordination sphere is presented as a plausible model based on the FeCl_3_ precursor and the observation of a chloro‐containing Fe–ligand adduct in the ESI–MS spectrum. However, the coordination sphere may also include solvent ligands (H_2_O/DMF), and definitive assignment will require additional structural verification. Fragmentation of this ion affords species consistent with loss of carboxyl‐derived moieties (fragments B, Figure S4). In addition, characteristic ligand‐derived ions are observed: H_2_L undergoes hydride elimination to give a signal at m/z 290.08 (fragment C), which further loses the carboxyl group to produce a fragment at m/z 246.09 (fragment D). Collectively, the presence of the Fe–ligand adduct ion and its fragmentation pathway, together with the ligand‐related fragments, is consistent with the formation of an Fe(III)–H_2_L coordination species under the measured conditions.

### 3.2. Solvent Selection and the Effect of Water on H_2_L Dissolution

The solubility profile of H_2_L in various solvents is presented in Figure S5a. The DMF:H_2_O (9:1, v/v) medium was selected based on solubility and signal‐preservation considerations. A preliminary solvent screen (AcN, DMF, DMSO, and MeOH) indicated that DMF provides the highest solubility of H_2_L and enables preparation of optically clear working solutions required for reproducible UV–Vis colorimetric measurements. Because sensing in purely organic media is less relevant to water analysis, the influence of water fraction was evaluated using DMF–water mixtures (Figure S5b). A composition containing 10% water (v/v) offered the best compromise between aqueous content and analytical performance, maintaining a clear yellow solution and preserving the characteristic absorption bands. At higher water contents, the absorption response was progressively attenuated, consistent with strong hydrogen‐bonding interactions that suppress the charge‐transfer contribution and reduce the measurable signal [31]. Although DMF is not a green solvent, reducing organic content and transitioning to greener aqueous co‐solvent systems (e.g., EtOH–water or other benign media) will be an important direction for future optimization.

UV–Vis analysis of H_2_L in the selected medium showed two characteristic absorption bands at *λ*_max_ = 365 nm (π–π^∗^) and *λ*_max_ = 441 nm (n–π^∗^), attributable to the conjugated aromatic framework and the imine (C=N) chromophore, respectively. Although the present DMF–water system provided a reliable optical response, the ligand solubility and overall practicality could be further improved by exploring alternative (greener) solvent systems, including binary or multicomponent mixtures, optimizing solvent ratios, and assessing chemical stability under storage conditions. Notably, the stability of the H_2_L solution in DMF–water was monitored for 2 weeks, during which no visible changes in color or apparent concentration were observed. Since Schiff bases can undergo hydrolysis of the imine bond in aqueous environments in a pH‐dependent manner, future study will systematically evaluate the long‐term stability of H_2_L (solid‐state storage and solution shelf life, including light‐ and temperature‐dependent effects), as well as the storage stability of the paper‐strip format and any signal drift in baseline and Fe^3+^ response after defined storage periods. In contrast, the L–Fe(III) complex exhibited reduced stability after a few days, as evidenced by visible precipitation; therefore, UV–Vis measurements should be implemented immediately after complex formation to ensure analytical accuracy and reliability.

### 3.3. Colorimetric Assay and pH Effects

The colorimetric response of H_2_L toward different metal ions was investigated in DMF:H_2_O (9:1, v/v) (Figure 4(a)). H_2_L solution is yellow, exhibiting bands at 365 and 441 nm due to π ⟶ π^∗^ and n ⟶ π transitions, corresponding to the aromatic ring and heteroatom lone pairs of electrons, respectively (Figure S5b). Upon reaction with various metal ions, only Fe(III) induced a significant redshift, resulting in a new broad peak at *λ*_max_ of 504 nm, indicating strong Fe(III) binding with H_2_L. This complexation triggered a color change from yellow to black (Figure 4(b)). No significant spectral or colorimetric changes were observed with other metal ions, reaffirming H_2_L’s selectivity for Fe(III) ions. Hence, upon addition of Fe^3+^, H_2_L exhibited an immediate visual response (yellow to black) after brief mixing. For consistency, UV–Vis spectra were recorded approximately 2 min after mixing, corresponding to the practical sampling time.

FIGURE 4(a) UV–Vis absorption spectra of various H_2_L–metal complexes (Zn^2+^, Fe^3+^, Cr^3+^, Ni^2+^, Mn^2+^, K^+^, Hg^2+^, Co^2+^, Mg^2+^, Ca^2+^, Ba^2+^, Pb^2+^, Na^+^, Al^3+^, and Cu^2+^) against H_2_L solution as the blank. (b) Color changes of H_2_L upon the addition of various metal ions. (c) The influence of pH on the formation of the L–Fe(III) complex, with color changes of L–Fe(III) at different pH values in the inset.

**Figure:**
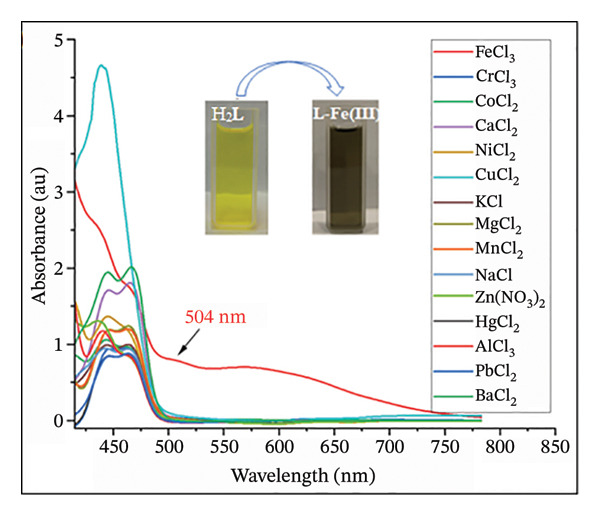
(a)

**Figure:**
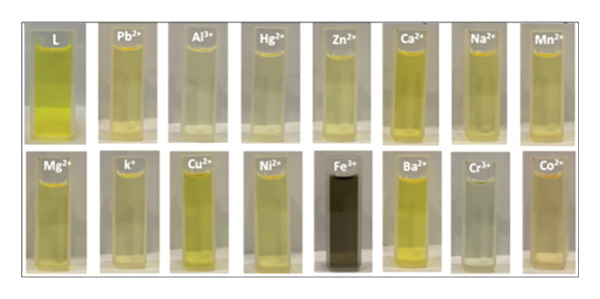
(b)

**Figure:**
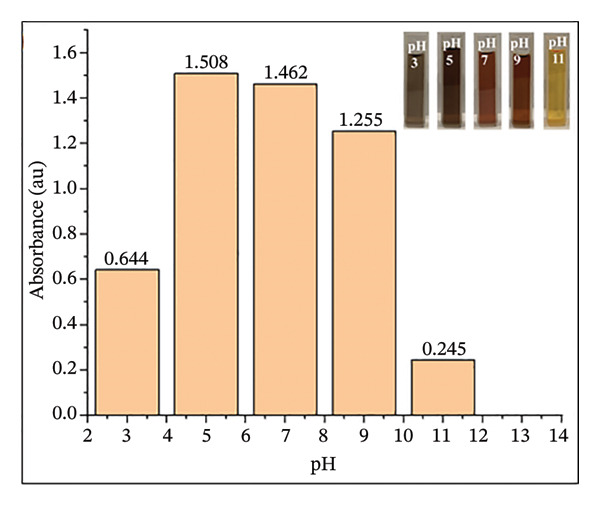
(c)

The effect of pH (3–11) on the absorption response of H_2_L to Fe(III) was also studied (Figure 4(c)). At pH = 3, the absorbance intensity of L–Fe(III) was low due to competition between Fe(III) and hydronium (H_3_O^+^) ions for H_2_L binding. As the pH increased to 5, the absorbance intensity increased due to enhanced electrostatic interactions between Fe(III) and deprotonated H_2_L. A color change from light black (pH = 3) to dark black (pH = 5) was observed (Figure 4(c), inset). At pH 7–9, the absorbance decreased slightly, and the black color became lighter due to Fe(OH)_3_ precipitation [52–54]. At pH 11, the solution turned yellow due to Fe(III) hydrolysis. Therefore, pH 5 was chosen as the most suitable for subsequent experiments. Although the selectivity of H_2_L was demonstrated against a broad panel of common cations relevant to water matrices, additional validation against Fe^2+^ and other less common trivalent ions (lanthanides such as La^3+^, Ce^3+^, Gd^3+^) would further strengthen the analytical scope. In aerated natural waters, Fe^2+^ is frequently oxidized to Fe^3+^, which is generally the more persistent and analytically relevant form under routine monitoring conditions. Nevertheless, future study should evaluate potential Fe^2+^ interference under controlled deoxygenated (and pH) conditions (to stabilize Fe^2+^) and assess possible competition from lanthanide ions across environmentally realistic concentration ranges and representative matrix compositions.

### 3.4. Binding Stoichiometry, Association Constant, and LOD

The binding stoichiometry between H_2_L and Fe(III) was counted via the Job plot method (Figure 5(a)). The absorbance intensity at *λ*_max_ = 504 nm peaked at a molar fraction of 0.5, confirming a 1:1 binding stoichiometry between H_2_L and Fe(III). This trend aligns with previously reported Fe(III) sensors [5, 13, 55, 56]. The proposed mechanism for the L–Fe(III) complex structure is illustrated in Figure 1. The association constant (ka) of L–Fe(III) was calculated as 2.87 × 10^4^ M^−1^ (Figure 5(b)), falling within the typical range of 10^3^–10^6^ M^−1^ for Fe(III)‐binding sensors [55–58]. The binding affinity was further examined using UV–Vis spectroscopy and colorimetric titration. As shown in Figure 5(c), a progressive increase in absorbance was observed with rising Fe(III) concentrations. Furthermore, a strong linear correlation (*R* ^2^ = 0.9963) was obtained between absorbance and Fe(III) concentration, demonstrating the reliability of the sensor’s response. The LOD was determined to be 3.71 μM, which is well below the WHO permissible limit for Fe(III) in drinking water, confirming the high sensitivity of H_2_L for Fe(III) detection (Figure 5(d)).

FIGURE 5(a) Job plot for the 1:1 complex of the Fe(III) ion and ligand H_2_L. (b) Benesi–Hildebrand plot for the formation of complex L–Fe(III), where [D_o_] is the ligand concentration and Abs is the absorbance of the complex at 504 nm. (c) Absorption spectra of H_2_L upon the addition of increasing concentrations of Fe(III) in DMF:H_2_O (9:1 vol/vol) using solvent–H_2_L solution as the blank. (d) Absorption intensity at 504 nm and pH = 5 as a function of the Fe(III) concentration.

**Figure:**
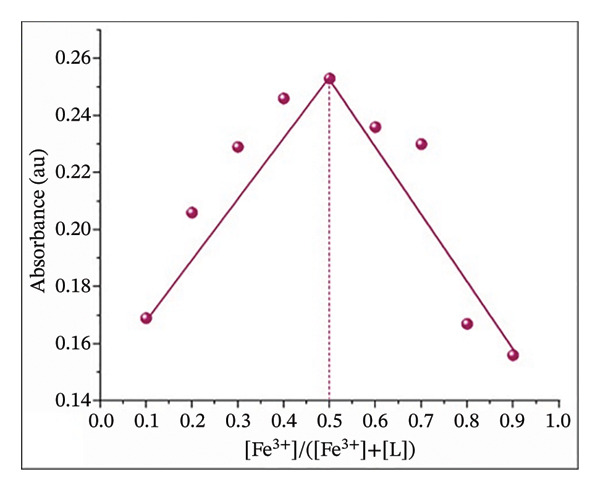
(a)

**Figure:**
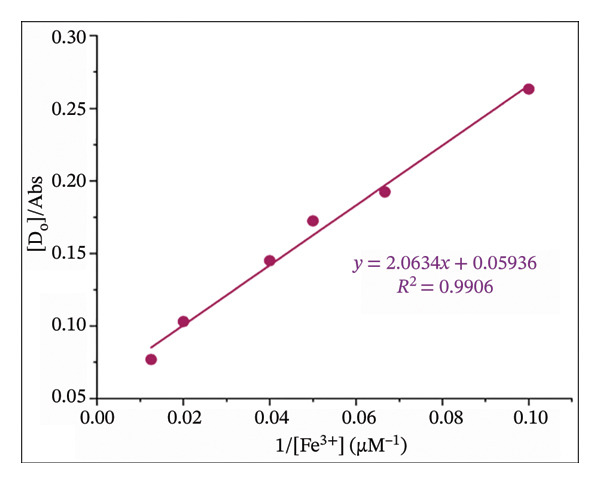
(b)

**Figure:**
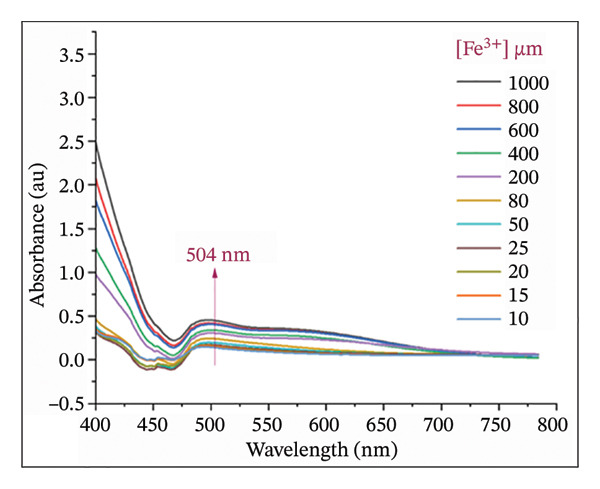
(c)

**Figure:**
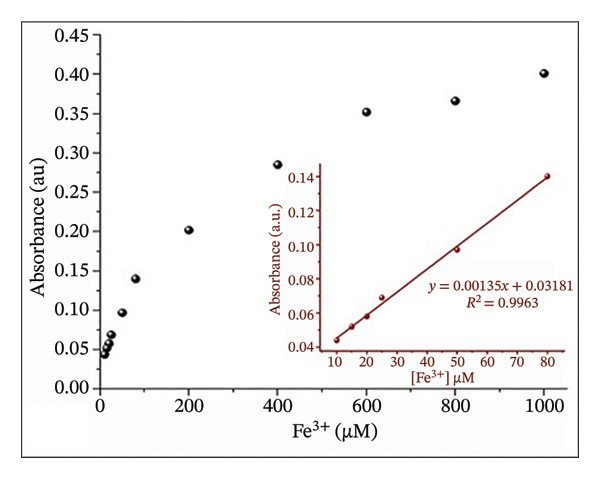
(d)

### 3.5. Competitive Studies on Real Water Samples

**FIGURE 6:**
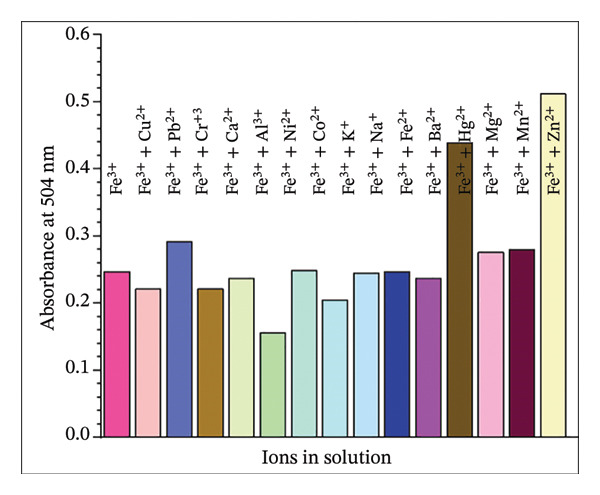
Changes in absorption intensity of H_2_L toward Fe(III) ion in the existence of the competing metal ions (Zn^2+^, Mn^2+^, Cr^3+^, Ni^2+^, K^+^, Hg^2+^, Co^2+^, Mg^2+^, Ca^2+^, Ba^2+^, Pb^2+^, Na^+^, Al^3+^, and Cu^2+^) at pH = 5.

The selectivity of the H_2_L sensor for Fe(III) was evaluated against various competing cations, including Al^3+^, Ba^2+^, Ca^2+^, Co^2+^, Cr^3+^, Cu^2+^, Hg^2+^, K^+^, Mg^2+^, Mn^2+^, Na^+^, Ni^2+^, Pb^2+^, and Zn^2+^. UV–Vis spectral analysis (Figure 6) revealed that the absorption profile of L–Fe(III) remained largely unaffected in the presence of other metal ions, demonstrating the exceptional selectivity of H_2_L for Fe(III). However, Hg^2+^, Zn^2+^, and Al^3+^ exhibited slight interference with Fe(III) complexation. The practical applicability of H_2_L for Fe(III) detection in real water samples was assessed using the spike and recovery approach, and the data are reported as mean ± standard deviation (*n* = 3) (Table 1). Water samples were spiked with known Fe(III) concentrations (100–300 μM), and the recovery percentages were calculated. The obtained recoveries ranged from 91.04% to 100.94%, confirming that H_2_L is an effective and reliable colorimetric chemosensor for Fe^3+^ detection in real environmental samples.

**TABLE 1:** Spike–recovery analysis for Fe(III) determination in distilled, tap, and dam water samples using the H_2_L colorimetric method.

Samples	Added ([Fe^3+^] μM)	Found ([Fe^3+^] μM)	% Recovery	RSD (%)
Distilled water	0	ND		
	100	99.12 ± 2.94	99.12 ± 2.94	2.97
	200	182.08 ± 4.97	91.04 ± 2.49	2.73
	300	278.38 ± 7.74	92.79 ± 2.58	2.78

Tap water	0	ND		
	100	94.67 ± 2.69	94.67 ± 2.69	2.84
	200	199.86 ± 5.98	99.93 ± 2.99	2.99
	300	302.82 ± 9.18	100.94 ± 3.06	3.03

Dam water	0	ND		
	100	96.90 ± 2.82	96.90 ± 2.82	2.91
	200	190.97 ± 5.42	95.49 ± 2.71	2.84
	300	274.67 ± 7.53	91.55 ± 2.51	2.74

*Note:* Results are reported as mean ± standard deviation; added concentration, found, recovery, and relative standard deviation (RSD %).

Abbreviation: ND, not detectable.

### 3.6. Detection of Fe(III) Using Paper Strips and Comparison With Other Sensors

After optimizing the reaction conditions in an aqueous media, a paper‐based assay was developed for Fe^3+^ detection. Paper strips were fabricated by cutting Whatman Grade 1 filter paper into appropriate dimensions and immersed in an H_2_L solution to achieve uniform coating. The coated strips were then air‐dried and used for Fe^3+^ sensing. Upon exposure to varying Fe^3+^ concentrations, the strips exhibited a noticeable color change from orange to brown, as shown in Figure 7(a) and Table S1. The color intensity was quantitatively analyzed using ImageJ software, which measured the mean intensity values. Results revealed a concentration‐dependent increase in color intensity over the range of 10–1000 μM, with an LOD of 50.7 μM (Figure 7(b)), lower than those reported for many previously developed Fe(III) sensors [58–60]. Consequently, this colorimetric paper strip technology provides a cost‐effective, rapid, and highly sensitive alternative for Fe^3+^ detection without requiring complex analytical instruments or procedures [47, 61]. As summarized in Table 2, the LOD achieved in this study surpasses that of several reported Fe(III) chemosensors. Additionally, the binding constant of the L–Fe(III) complex was higher than those reported in most earlier studies [62–67], further highlighting the enhanced sensitivity and efficiency of H_2_L as a reliable chemosensor for Fe(III) detection.

FIGURE 7(a) Photographs of H_2_L test strips after exposure to Fe(III) solutions with increasing molar concentrations. (b) Calibration plot of H_2_L test strips treated with various Fe(III) concentrations at pH = 5; color intensity (arbitrary unit, au) as analyzed by ImageJ software.

**Figure:**
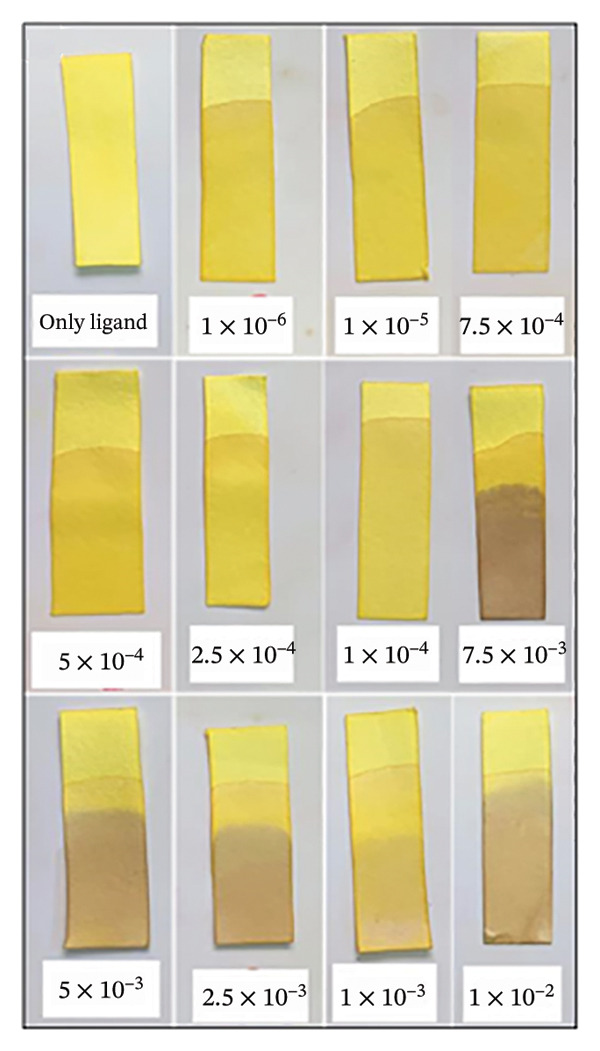
(a)

**Figure:**
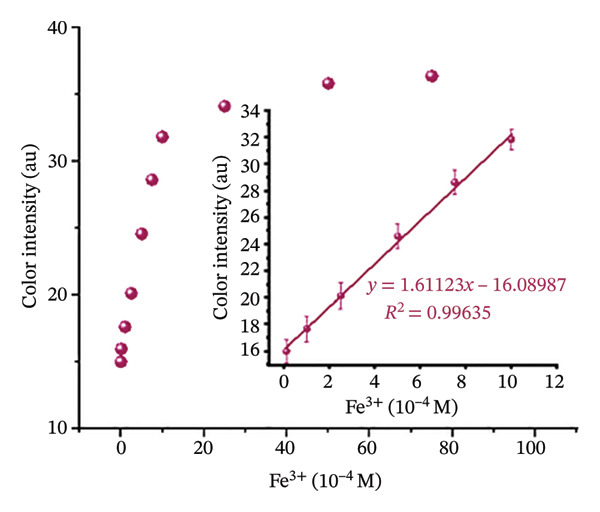
(b)

**TABLE 2:** Comparison of Fe(III) detection with other chemosensors.

Chemosensor ligand	Detection limit (M)	Binding stoichiometry	Binding constant (M^−1^)	Testing media (v/v)	Ref.
Chemosensor (probe 1)	5.38 × 10^−6^	1:1	0.94 × 10^4^	EtOH:H_2_O (8:2, v/v)	[54]
Chemosensor quercetin	20 × 10^−6^	1:1	—	DMSO:H_2_O (9:1, v/v)	[55]
Rhodamine‐based dual sensor	1 × 10^−4^	1:1	1.65 × 10^3^	75% CH_3_CN 25% 0.01 M Tris–HCl buffer	[56]
Chemosensor 1	13.5 × 10^−6^	1:1	—	DMF–buffer solution (1:1, v/v)	[57]
Chemosensor SL1	1 × 10^−5^	1:2	—	DMF	[58]
Chemosensor RDFB	6.94 × 10^−6^	1:1	1.13 × 10^5^	CH_3_CN–aq. HEPES buffer medium.	[59]
Chemosensor H_2_L	3.37 × 10^−6^	1:1	2.87 × 10^4^	DMF/water (9:1, v/v)	This study

The performance and applicability of the H_2_L chemosensor may be further improved through structural modifications, optimization of sensing conditions, or even adopting alternative detection techniques. Although H_2_L exhibits high selectivity toward Fe^3+^ with minimal interference from the tested ions (a key advantage), targeted chemical modifications, such as substituting the phenolic hydroxyl or other coordination sites with alternative chelating functionalities (e.g., ‐SH, ‐NH_2_, or ‐COOH), could modulate the coordination environment and potentially broaden its metal ion selectivity or sensitivity. In addition, sensing parameters such as solvent system, sensing medium, and pH can substantially influence the coordination efficiency and overall response. While the Fe^3+^‐induced color change is visually instantaneous, a dedicated time‐resolved study (seconds–minutes) should be performed in future study to quantitatively determine the response time and to evaluate kinetic behavior across different pH values and complex sample matrices. Finally, beyond solution‐based assays, emerging formats such as paper‐based sensors, microfluidic devices, and smartphone‐assisted readout offer portable and instrument‐free alternatives for on‐site analysis; therefore, future study should focus on the development of a microfluidic paper‐based analytical device for rapid, visual, and quantitative field detection.

## 4. Conclusion

In conclusion, a new Schiff base–derived colorimetric chemosensor (H_2_L), obtained from 2‐hydroxy‐1‐naphthaldehyde and anthranilic acid, was developed and validated for selective Fe^3+^ detection. In a semi‐aqueous DMF:H_2_O (9:1, v/v) medium, H_2_L exhibited a distinct and selective response to Fe^3+^ among common competing cations, producing an obvious yellow‐to‐black color change and a new broad absorption band in the visible region (504 nm). Binding studies confirmed a 1:1 stoichiometry with an association constant of 2.87 × 10^4^ M^−1^, and the method achieved a low UV–Vis detection limit of 3.71 μM. The sensing performance was pH‐dependent, with optimum response at pH 5, while higher pH values diminished the signal due to iron hydrolysis. Practical applicability was demonstrated through spike–recovery analysis in distilled, tap, and dam water (recoveries 91.04%–100.94%) and a paper‐strip format coupled with image‐smartphone‐based readout, enabling rapid on‐site screening. Overall, these findings support H_2_L as a simple and effective platform for Fe^3+^ monitoring in water‐relevant matrices, and they motivate future study toward greener sensing media, time‐resolved response profiling, and broader selectivity validation.

## Funding

The authors declare no funding for this research.

## Conflicts of Interest

The authors declare no conflicts of interest.

## Supporting Information

Table S1: Data of H_2_L test strips dipped in various concentrations of Fe(III).

Figure S1: Schematic illustration for paper strip fabrication coated with chemosensor H_2_L for the colorimetric test of Fe^3+^.

Figure S2: (a) Images (top) and color inverted (bottom), (b) image analyzed using ImageJ free software through three channels (blue, green, and red) by ticking on “image” and choosing “color” and selecting “split channel,” (c) color intensity of the reaction zone was obtained by clicking on the “measure” tab under “analyze.”

Figure S3: Mass fragmentation profile of (a) H_2_L and (b) L–Fe(III) complex.

Figure S4: Schematic illustration of L–Fe(III) complex possible mass fragments.

Figure S5: Effect of solvent on solubility of H_2_L (a) and UV–Vis spectra of chemosensors H_2_L upon the influence of the DMF/H_2_O ratio on the dissolution of prepared ligand (b).

## Supporting information


**Supporting Information** Additional supporting information can be found online in the Supporting Information section.

## Data Availability

The datasets used are available from the corresponding author upon reasonable request.
