# Supplementary material for: Schiff Base–Derived Colorimetric Chemosensor for Fe3+ Detection: Synthesis, Mechanistic Insights, and Real‐World Applications in Water Quality Monitoring
Source: Int J Anal Chem. 2026 Feb 23;2026:3977293. doi: 10.1155/ianc/3977293 (PMC12927900; doi:10.1155/ianc/3977293)
Supplement: Supplementary file 1 — Supporting Information Additional supporting information can be found online in the Supporting Information section. [file IANC-2026-3977293-s001.docx]

**Supplementary Information**

**Schiff Base-Derived Colorimetric Chemosensor for Fe³⁺ Detection: Synthesis, Mechanistic Insights, and Real-World Applications in Water Quality Monitoring**

Abstract:

A Schiff base colorimetric chemosensor (H_2_L), synthesized from 2-hydroxy-1-naphthaldehyde and anthranilic acid, was developed for selective Fe^3+^ detection. In a DMF:H₂O (9:1, v/v) medium, H₂L exhibited a distinct and selective response to Fe^3+^ among common competing cations, producing a visible color change from yellow to black, and a new broad absorption band at 504 nm. Job’s plot indicated a 1:1 binding stoichiometry, and the association constant was 2.87 × 10^4^ M^−1^. The sensor showed a low detection limit of 3.71 µM by UV–Vis titration, and its practical applicability was validated by spike–recovery analysis in real water samples (recoveries 91.04–100.94%). A paper-strip assay enabled rapid on-site screening with smartphone image analysis, achieving an LOD of 50.7 µM. These results highlight H_2_L as a simple and effective platform for Fe^3+^ monitoring in water-relevant matrices.

*Keywords*: binding stoichiometry; colorimetric detection; Fe³⁺ detection; paper strip detection; Schiff base

**Table S1**: Data of H_2_L-test strips dipped in various concentrations of Fe(III).

**Figure S1**: Schematic illustration for paper strip fabrication coated with chemosensor H_2_L for the colorimetric test of Fe^3+^

**Figure S2**: (a) images (Top) and color inverted (Bottom), (b) Image analyzed using ImageJ free software through three channels (blue, green, Red) by ticking on “image” and choosing “color” and selecting “spilt channel”, (c) Color intensity of reaction zone was obtained by clicking on " measure" tab under "analyze"

**Figure S3**: Effect of solvent on solubility of H_2_L (a) and UV–Vis spectra of chemosensors H_2_L upon the influence of the DMF/H2O ratio on the dissolution of prepared ligand (b)

**Figure S4**: Mass fragmentation profile of (a) H_2_L and (b) L-Fe(III) complex

Figure S5: Schematic illustration of possible mass fragmentation process

**Table S1:** Data of H_2_L-test strips dipped in various concentrations of Fe(III).

| **[Fe(III) x10^-4^ M]** | **Average intensity** | **Standard deviation(SD)** | **RSD%** |
| --- | --- | --- | --- |
| 100 | 54.63 | 0.87 | 1.59 |
| 75 | 36.38 | 0.60 | 1.65 |
| 50 | 35.83 | 0.70 | 1.95 |
| 25 | 34.13 | 0.94 | 2.75 |
| 10 | 31.83 | 0.76 | 2.39 |
| 7.5 | 28.63 | 0.95 | 3.31 |
| 5 | 24.58 | 0.91 | 3.70 |
| 2.5 | 20.13 | 0.98 | 4.86 |
| 1 | 17.63 | 0.95 | 5.39 |
| 0.1 | 15.98 | 0.88 | 5.51 |
| 0.01 | 15.03 | 0.75 | 4.99 |


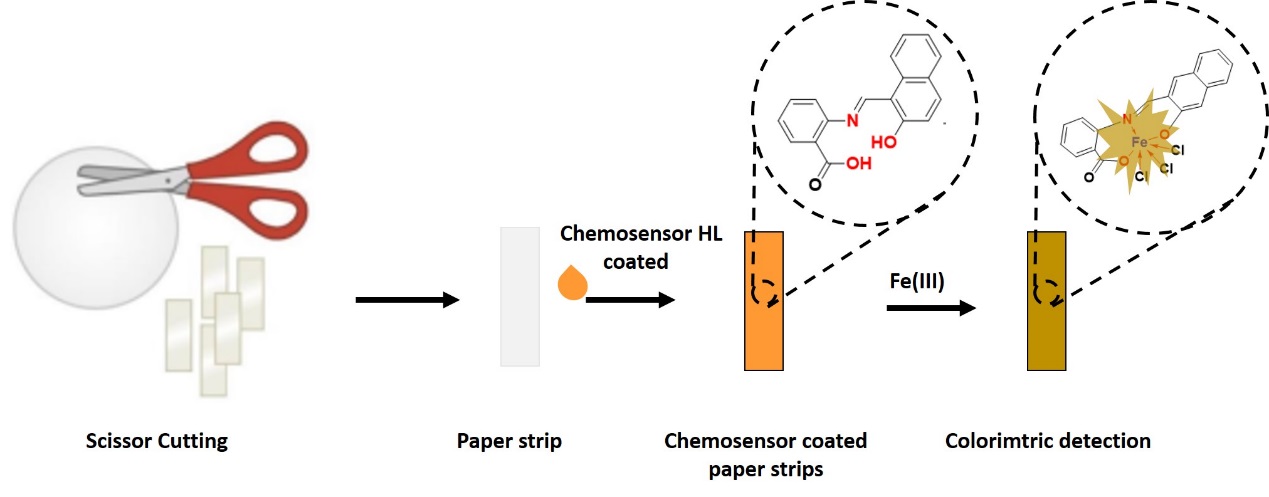


**Figure S1:** Schematic illustration for paper strip fabrication coated with chemosensor H_2_L for the colorimetric test of Fe(III)


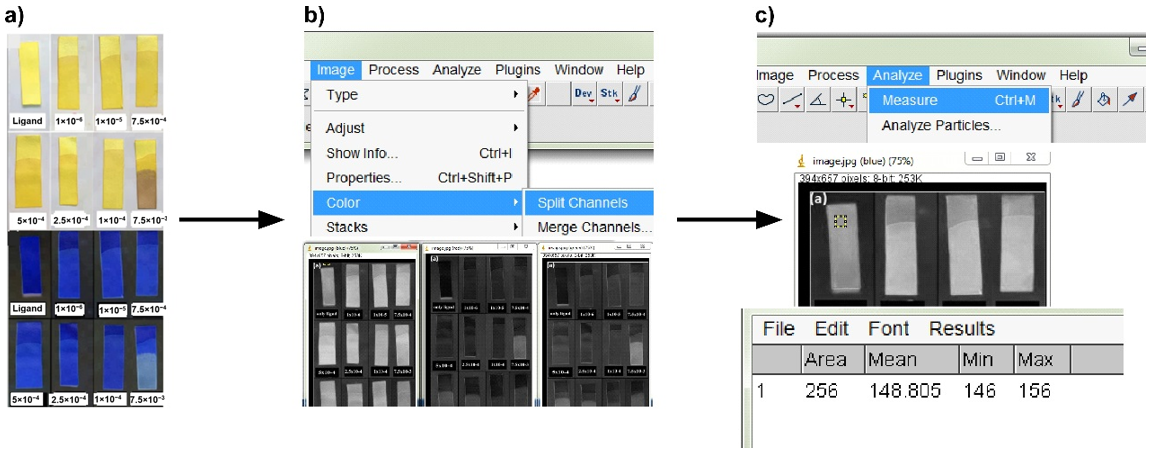


**Figure S2:** (a) images (Top) and color inverted (Bottom), (b) Image analyzed using ImageJ free software through three channels (blue, green, Red) by ticking on “image” and choosing “color” and selecting “spilt channel”, (c) Color intensity of reaction zone was obtained by clicking on " measure" tab under "analyze"


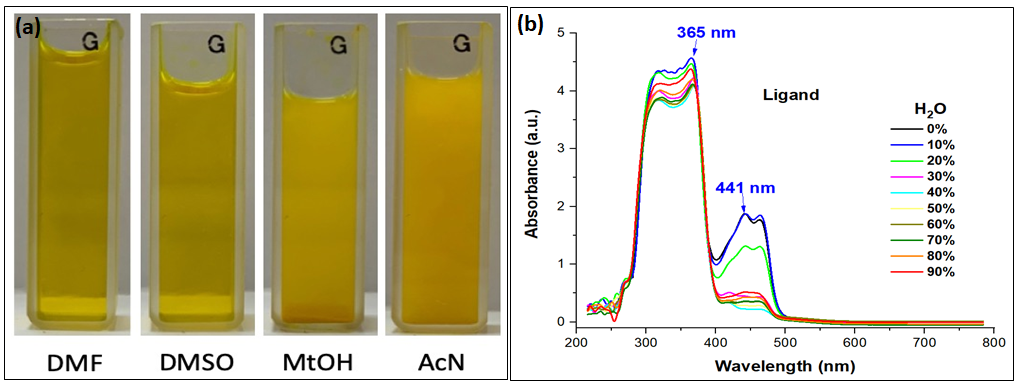


Figure S3: Effect of solvent on solubility of H_2_L (a) and UV-Vis spectra of chemosensors H_2_L upon the influence of the DMF/H_2_O ratio on the dissolution of prepared ligand (b)


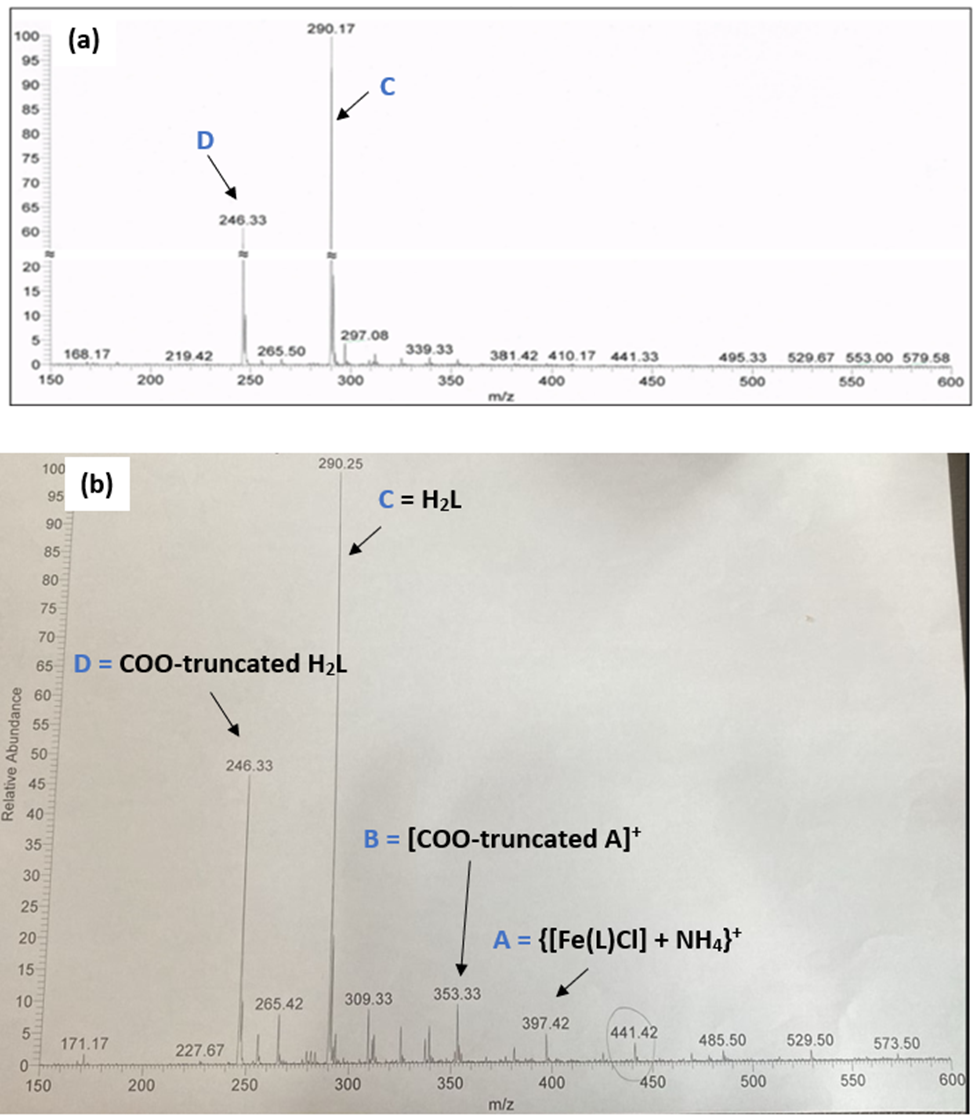


Figure S4 Mass fragmentation profile of (a) H_2_L and (b) L-Fe(III) complex

Figure S5: Schematic illustration of L-Fe(III) complex possible mass fragments
